# Characterization of serotonin-induced inhibition of excitatory synaptic transmission in the anterior cingulate cortex

**DOI:** 10.1186/s13041-017-0303-1

**Published:** 2017-06-12

**Authors:** Zhen Tian, Manabu Yamanaka, Matteo Bernabucci, Ming-gao Zhao, Min Zhuo

**Affiliations:** 1 Center for Neuron and Disease, Frontier Institutes of Science and Technology, Xi’an Jiaotong University, Xi’an, Shanxi 710049 China; 2 Department of Pharmacy, The 154th central hospital of PLA, Xinyang, Henan 464000 China; 3 Department of Physiology, Faculty of Medicine, University of Toronto, 1 King’s College Circle, Toronto, ON M5S 1A8 Canada

**Keywords:** Anterior cingulate cortex, Serotonin, Excitatory postsynaptic currents, Adenylyl cyclase, Glutamatergic neurotransmission

## Abstract

Excitatory synaptic transmission in central synapses is modulated by serotonin (5-HT). The anterior cingulate cortex (ACC) is an important cortical region for pain perception and emotion. ACC neurons receive innervation of projecting serotonergic nerve terminals from raphe nuclei, but the possible effect of 5-HT on excitatory transmission in the ACC has not been investigated. In the present study, we investigated the role of 5-HT on glutamate neurotransmission in the ACC slices of adult mice. Bath application of 5-HT produced dose-dependent inhibition of evoked excitatory postsynaptic currents (eEPSCs). Paired pulse ratio (PPR) was significantly increased, indicating possible presynaptic effects of 5-HT. Consistently, bath application of 5-HT significantly decreased the frequency of spontaneous and miniature excitatory postsynaptic currents (sEPSCs and mEPSCs). By contrast, amplitudes of sEPSCs and mEPSCs were not significantly affected. After postsynaptic application of G protein inhibitor GDP-β-S, 5-HT produced inhibition of eEPSCs was significantly reduced. Finally, NAN-190, an antagonist of 5-HT_1A_ receptor, significantly reduced postsynaptic inhibition of 5-HT and abolished presynaptic inhibition. Our results strongly suggest that presynaptic as well as postsynaptic 5-HT receptor including 5-HT1A subtype receptor may contribute to inhibitory modulation of glutamate release as well as postsynaptic responses in the ACC.

## Introduction

As one important neurotransmitter in the central nervous system (CNS), serotonin (5-hydroxytryptamine, 5-HT) plays a crucial role in numerous physiological functions. It has been implicated in pain modulation [1] and emotional disorders [2]. Serotonergic projections to spinal or medullary dorsal horn areas are involved in nociceptive transmission through regulating descending inhibitory pathways [3–5]. Mice lacking serotoninergic neurons in the CNS exhibited enhanced inflammation pain and reduced analgesia in response to opioids and antidepressants [6]. The intrathecal administration of serotonergic agents induced anti-nociceptive actions in animal models [7]. A recent study demonstrates that activation of a specific 5-HT subtype receptor inhibited mechanical allodynia in nerve-injured animals by affecting hyperpolarization-activated cyclic nucleotide-regulated (HCN) channels dendritic function in the anterior cingulate cortex (ACC) [8].

The ACC is a critical cortical region, which plays a central role in the formation of pain perception and the unpleasantness of pain [1, 9, 10]. Neurons of the ACC receive sensory inputs projecting from other subcortical areas (such as the thalamus) and then project to related sensory regions, including amygdala, midbrain areas, brainstem and spinal cord [11, 12]. Injuries enhanced synaptic transmission in the ACC and inhibition of the ACC potentiation is analgesic in animal models of chronic pain [1, 9, 10]. In the CNS, serotoninergic neurons are mainly located in the raphe nuclei, and they send projecting terminals to different regions of the brain and spinal cord [13]. At the synaptic level, 5-HT has been reported to produce only inhibitory or biphasic modulation of sensory synaptic transmission. For example, 5-HT exerts a biphasic modulation of glutamate transmission in the dorsal horn of the spinal cord [14, 15]. 5-HT produces only inhibitory effects on excitatory or inhibitory transmission in the bed nucleus of the stria terminalis (BNST) [16] and the periaqueductal grey (PAG) [17]. In forebrain areas including the ACC, neurons are highly innervated by serotoninergic terminals [13, 18]. However, the possible effect of 5-HT on excitatory synaptic transmission in the ACC has not been investigated. In the present study, we perform whole-cell patch-clamp recordings from ACC pyramidal cells and investigate the modulatory effect of 5-HT. We found that bath application of 5-HT produced only inhibitory modulation of excitatory synaptic transmission. Furthermore, we show that 5-HT may produce its inhibitory effects through both presynaptic and postsynaptic mechanisms.

## Methods

### Animals

Adult male C57BL/6 mice (8–14 weeks) were used in the experiments and they were purchased from Charles River Laboratories (St. Constant, Quebec, Canada). The animals were housed in plastic boxes with food and water available ad libitum in a colony room with controlled temperature (24 ± 2 °C), humidity (50–60%), and a 12:12 h light-dark cycle. Experiments were performed under protocols approved by the Animal Care and Use Committee at the University of Toronto.

### Chemicals

5-HT and guanosine-5′-O-(2-thiodiphosphate) (GDP-β-S) were obtained from Sigma–Aldrich (St. Louis, MO, USA). NAN-190, picrotoxin and tetrodotoxin were purchased from Tocris Cookson (Bristol, UK). Drugs were prepared as stock solutions for frozen aliquots at −20 °C. All these drugs were diluted from the stock solution to the final desired concentration in the artificial cerebrospinal fluid (ACSF) before immediate use. All of other chemicals and reagents used were commercially available and of standard biochemical quality.

### Brain slice preparation

The general procedures for making the ACC slices were similar to those described previously [19, 20]. Mice were anesthetized with isoflurane in air and then decapitated. Brains were rapidly removed and placed for 2–3 min in an ice-cold and oxygenated ACSF (in mM): containing 124 NaCl, 25 NaHCO_3_, 2.5 KCl, 1 KH_2_PO_4_, 2 CaCl_2_, 2 MgSO_4_ and 10 glucose, and continuously gassed with 95% O_2_/5% CO_2_. Coronal slices (300 μm) containing the ACC were prepared on a vibratome (Leica VT1200S) in ice-cold ACSF. Slices were then incubated in a room temperature-submerged recovery chamber with oxygenated (95% O_2_ and 5% CO_2_) ACSF for at least 1 h.

### Whole-cell patch-clamp recording

After recovery, slices were placed in a recording chamber on the stage of an Olympus microscope with infrared digital interference contrast optics for visualization of whole-cell patch-clamp recordings. Recordings were performed at room temperature (21–23 °C) with continuous perfusion of ACSF at a rate of 2 mL/min. For spontaneous excitatory postsynaptic currents (sEPSCs) recording, recording pipettes (3–5 MΩ) were filled with solution containing 145 mM K-gluconate, 5 mM NaCl, 1 mM MgCl_2_, 0.2 mM EGTA, 10 mM HEPES, 2 mM Mg-ATP, and 0.1 mM Na_3_-GTP, adjusted to pH 7.2 with KOH (280–300 mOsm). sEPSCs were collected in the neurons clamped at −60 mV in the ACSF. For the recording of miniature excitatory postsynaptic currents (mEPSCs), the circulating ACSF was additionally added 1 μM tetrodotoxin. The evoked EPSCs (eEPSCs) were recorded from layer II/III neurons with an Axon 200B amplifier (Molecular Devices) and stimulation was delivered by a bipolar tungsten-stimulating electrode placed in layer V/VI of the ACC. The internal solution containing GDP-β-S was used when the experiment to detect the involvement of postsynaptic G proteins was performed. For the recording of paired-pulse ratio (PPR), a paired pulse paradigm was employed in which two stimuli were delivered at 50 ms inter-stimulus-interval. Picrotoxin (100 μM) was always present to block GABA_A_ receptor-mediated inhibitory synaptic currents in all experiments. Access resistance (15–30 MΩ) was monitored throughout the experiment. Data were discarded if access resistance changed >15% during an experiment.

#### Data analysis

Whole-cell patch-clamp data were collected and analyzed with Clampex 10.2 and Clampfit 10.2 software (Molecular Devices). For the evoked EPSCs, the amplitudes were normalized and expressed as the percentage of the baseline EPSC amplitude. Miniature and spontaneous EPSCs were detected and analyzed using an event detection program (Mini Analysis Program; Synaptosoft, Inc., Decatur, GA). Analysis of mEPSCs and sEPSCs was performed with cumulative probability plots. For the PPR, the ratio of the slope of the second response to the slope of the first response was calculated and averaged. For comparison between two groups, we used paired or unpaired Student’s *t* test. For comparison among three groups, we used one-way ANOVA. All data are presented as means ± SEM. In all cases, *p* < 0.05 was considered statistically significant.

## Results

### Effect of 5-HT on excitatory transmission in the ACC

**Fig. 1:**
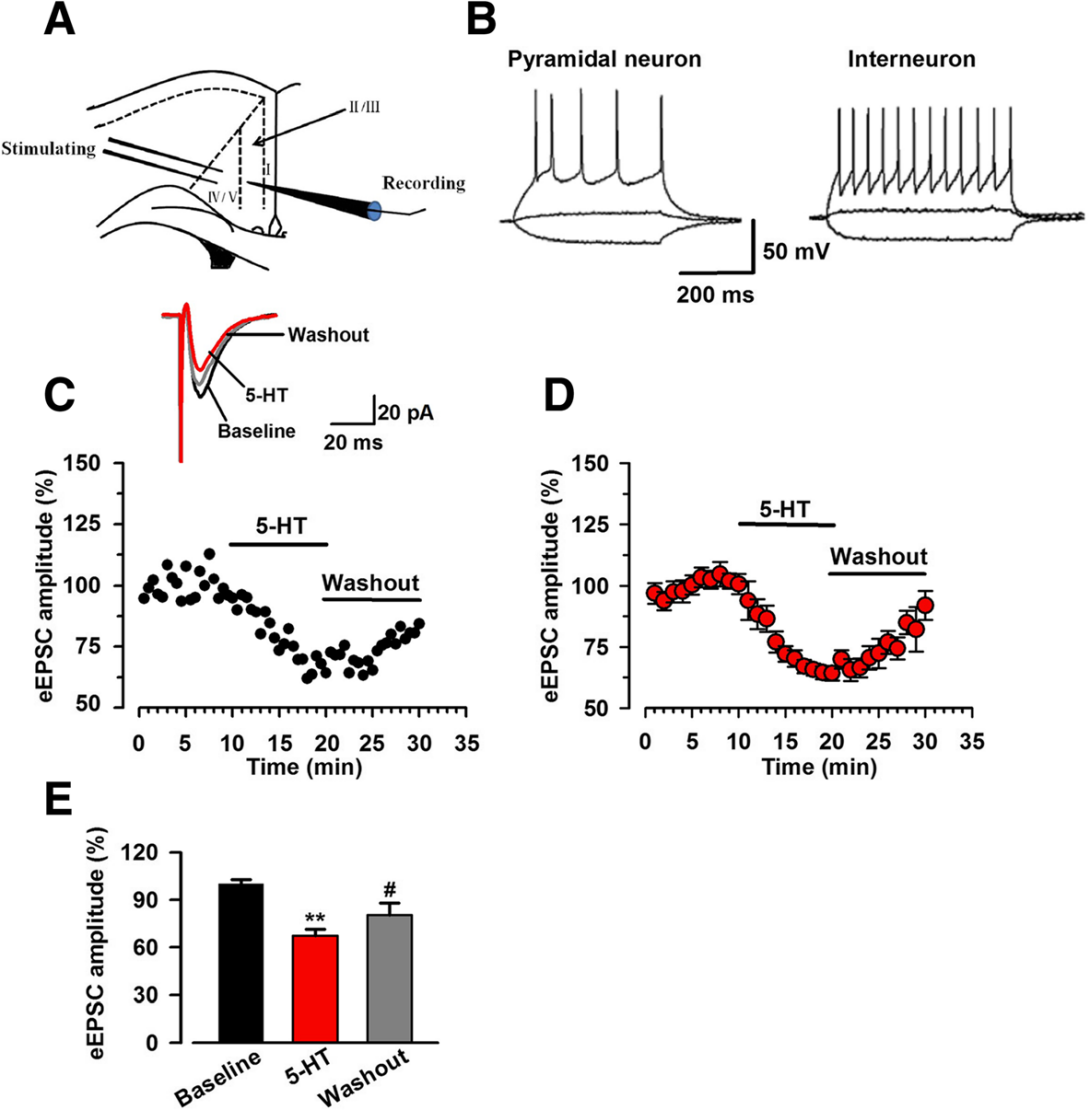
Bath application of 5-HT reduced the amplitude of evoked EPSC. **a** Schematic diagram of a slice illustrating the placement of a whole-cell patch recording and stimulating electrode in anterior cingulate cortex. **b** Current-clamp recordings to identify pyramidal neurons (*left*) and interneurons (*right*) by step current injection. **c** An example showing the time course of recorded pyramidal neurons in layer II/III of ACC after application of 5-HT (5 μM) and washout. The insets showed averaged trace of six eEPSCs about baseline, application of 5-HT and washout. **d** The averaged time course of recorded neurons (*n* = 9 neurons/6 mice) in ACC. Bath application of 5-HT (5 μM) led to a gradual reduction of eEPSC amplitude and washing out 5-HT with ACSF caused the rise of eEPSC amplitude again. **e** Summary of the effect of 5-HT (5 μM) and washout on the amplitude of eEPSC. Bath application of 5-HT significantly reduced eEPSCs amplitude and washing the system with fresh ACSF partially but evidently reversed the decreased eEPSC (*n* = 9 neurons/6 mice). ^**^ *p* < 0.01 compared to baseline; ^#^ *p* < 0.05 compared to the group of 5-HT

To explore the effect of 5-HT on the glutamatergic neurotransmission in the ACC, we performed whole-cell patch-clamp recordings from pyramidal neurons in layer II/III, where the stimulation electrodes were placed in layer V/VI (Fig. 1a). To identify pyramidal neurons, depolarized currents were injected into neurons to induce action potentials. The typical firing pattern of pyramidal neurons exhibited as significant firing frequency adaptation in response to the prolonged depolarizing-current injection [21], whereas interneurons showed fast-spiking action potentials followed by pronounced hyperpolarization [19] (Fig. 1a). In the presence of the GABA_A_ receptor antagonist, picrotoxin (100 μM), EPSCs evoked by a single-pulse stimulation were recorded with the membrane potential holding at −60 mV and the baseline amplitude of eEPSCs was adjusted at 50–100 pA. After obtaining stable baseline eEPSCs for at least 10 min, we bath applied 5-HT (5 μM or 50 μM) for 10 min and then washed the system with oxygenated ACSF for another 10 min. The amplitude of evoked EPSCs were significantly decreased after bath application of 5-HT in a dose-dependent manner (5 μM: 67.3 ± 4.2% of baseline, *n* = 9 neurons/6 mice, *p* < 0.01, see Fig. 1c, d, e; 50 μM: 50.6 ± 8.7% of baseline, *n* = 6/4, *p* < 0.01, data not shown), indicating that glutamatergic transmission in the ACC was inhibited by 5-HT. Washing out of 5-HT with control ACSF, it partially reversed the reduction of eEPSC amplitude caused by 5-HT (5 μM: 80.5 ± 7.4% of baseline, *n* = 9/6, *p* < 0.05 compared with the 5-HT, Fig. 1d and e; 50 μM: 66.1 ± 6.3% of baseline, *n* = 6/4, data not shown).

### Altered paired-pulse ratio by 5-HT

**Fig. 2:**
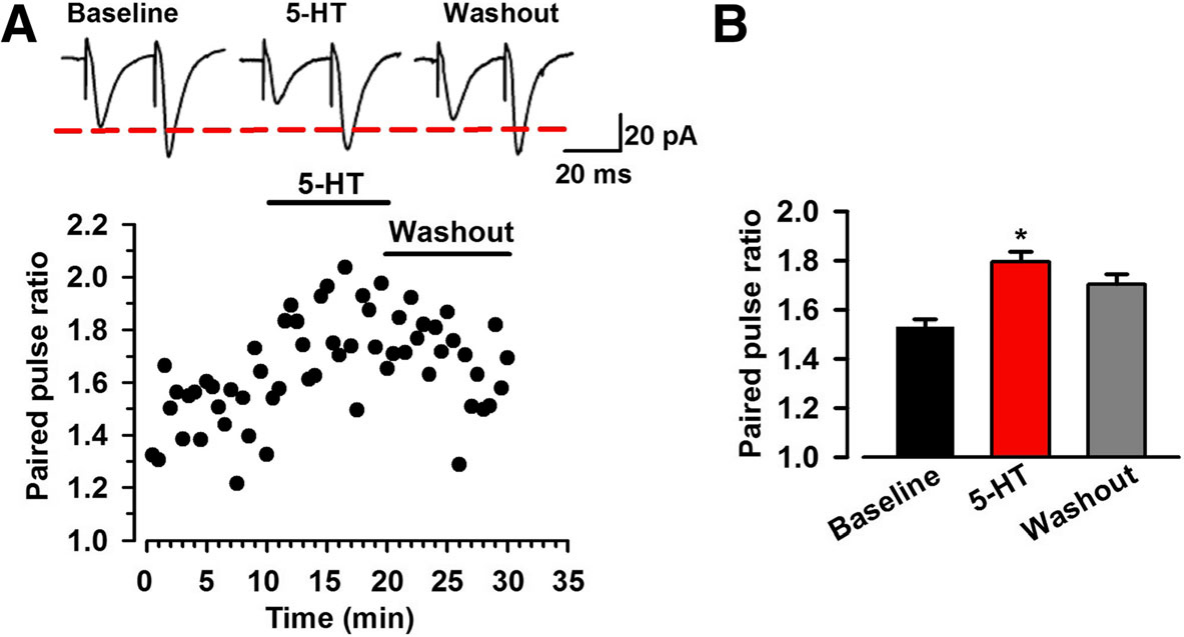
Bath application of 5-HT increased paired-pulse ratio. **a** Paired-pulse ratio (the ratio of EPSC2/EPSC1) was recorded with a 50 ms interval. One example showing the time course of PPR recorded in the ACC layer II/III neurons. The insets showed averaged trace of six sweeps about baseline, application of 5-HT and washout.**b** Summary of the effect of 5-HT (5 μM) and washout on the PPR. 5-HT increased the PPR notably (*n* = 10 neurons/7 mice). ^*^ *p* < 0.05 compared to baseline

To investigate whether presynaptic or postsynaptic mechanisms mediate the effects of 5-HT on excitatory synaptic transmission in the ACC, we examined PPR in ACC. An alteration of PPR reflects the change in release probability from presynaptic terminals. Bath application of 5-HT (5 μM) significantly increased the PPR at a stimulus interval of 50 ms in most of recording ACC pyramidal neurons (baseline 1.53 ± 0.08; 5-HT 1.79 ± 0.11, *n* = 10 of 12 neurons from 7 mice, *p* < 0.05; Fig. 2a and b; the other two neurons: one had no remarkable change, the other decreased from 1.41 to 1.19). This finding indicates that the presynaptic mechanism is involved in 5-HT-mediated inhibition of excitatory synaptic transmission in the ACC.

### Effect of 5-HT on sEPSC in the ACC

**Fig. 3:**
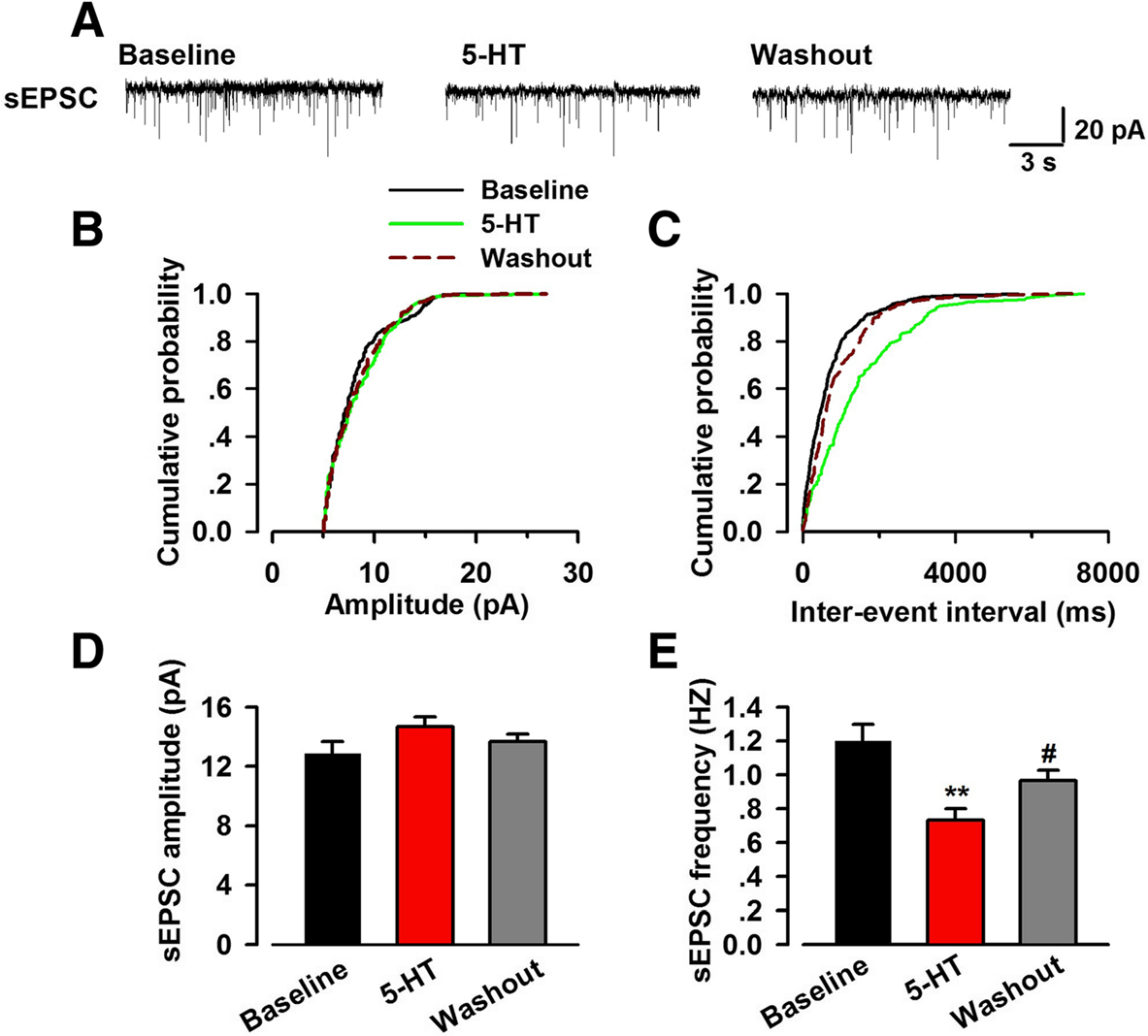
Effect of 5-HT on sEPSC recorded in neurons of the ACC. **a** Representative trace of sEPSC recorded in pyramidal neurons of the ACC layer II/III at a holding potential of −60 mV. **b** Cumulative probability plot showing the distribution of sEPSC amplitude in the phase of baseline, 5-HT application (5 μM) and washout.**c** Cumulative inter-events interval plot of recorded sEPSC in the phase of baseline, 5-HT application (5 μM) and washout. Black fill line indicated the phase of baseline, green fill line indicted the phase of 5-HT application and deep red dashed line indicated the phase of washout. **d** Summary result of averaged sEPSC amplitude (*n* = 15 neurons/7 mice). (E) Summary result of averaged sEPSC frequency (*n* = 12 neurons/7 mice). 5-HT (5 μM) application significantly reduced the frequency of sEPSC and washout partially reversed the reduction. ^**^ *p* < 0.01 compared to baseline; ^#^ *p* < 0.05 compared to the phase of 5-HT application

Spontaneous events are thought to be the results of the presynaptic action potential evoked neurotransmitter vesicles release from the readily releasable pool [22]. The effects of 5-HT on sEPSC in the pyramidal neurons of ACC were examined. As shown in Fig. 3, the frequency of sEPSC was significantly decreased in most of the recorded neurons with the bath application of 5-HT (baseline 1.20 ± 0.10 Hz; 5-HT, 0.73 ± 0.07 Hz, *n* = 12 of 15 neurons/7 mice, *p* < 0.01; Fig. 3a, c, e; the other 3 neurons did not change significantly). A cumulative fraction plot showed an evident increase of inter-event-interval during 5-HT application (Fig. 3c). In those responded neurons, following washout with ACSF, the decreased sEPSC frequency could be reversed partly but not totally (5-HT 0.73 ± 0.07 Hz, washout 0.97 ± 0.06 Hz, *n* = 12/7, *p* < 0.01; Fig. 3a, c, e). The amplitude of sEPSC was not affected by the application of 5-HT or washout with control ACSF (baseline 12.9 ± 0.8 pA, 5-HT 14.7 ± 0.6 pA, washout 13.7 ± 0.5, *n* = 15/7; Fig. 3a, b, d).

### Effect of 5-HT on mEPSC

**Fig. 4:**
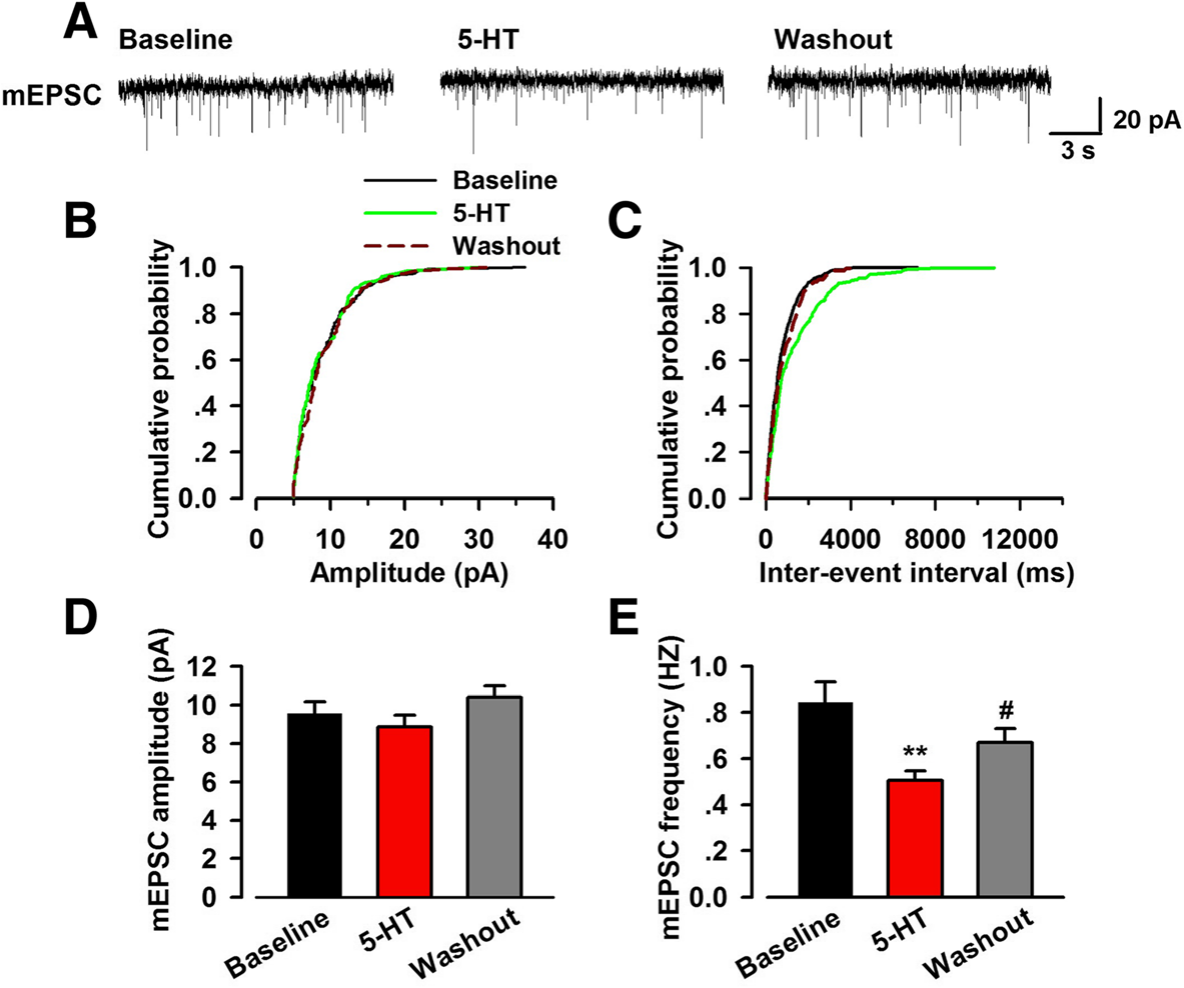
Effect of 5-HT on mEPSC recorded in neurons of the ACC. **a** Representative mEPSC recorded in pyramidal neurons of the ACC layer II/III at a holding potential of −60 mV. **b** Cumulative plot of mEPSC amplitude of the phase of baseline, 5-HT application (5 μM) and washout. **c** Cumulative inter-event interval plot of recorded mEPSC in the phase of baseline, 5-HT application (5 μM) and washout. Black fill line indicated the phase of baseline, green fill line indicted the phase of 5-HT application and deep red dashed line indicated the phase of washout. **d** Summary plots of mEPSC amplitude (*n* = 12 neurons/6 mice). **e** Summary plots of mEPSC frequency (*n* = 10 neurons/6 mice). 5-HT (5 μM) application significantly reduced the frequency of mEPSC and washout partially reversed the reduction. ^**^ *p* < 0.01 compared to baseline; ^#^ *p* < 0.05 compared to the phase of 5-HT application

Miniature synaptic transmission is resulted from neurotransmitter release independent of action potential [23], which occurs randomly in the absence of stimuli [24]. We recorded mEPSCs in the ACC pyramidal neurons in the presence of 1.0 μM tetrodotoxin to further determine the role of presynaptic mechanisms in the inhibitory effects of 5-HT. We found that mEPSC frequency in most of the recorded neurons were reduced after perfusing 5-HT (baseline 0.84 ± 0.10 Hz; 5-HT, 0.52 ± 0.05 Hz, *n* = 10 of 12/6, *p* < 0.01; Fig. 4a, c, e; the other 2 neurons: one increased from 0.68 to 0.80, one had no remarkable change). A cumulative fraction plot showed an increase of inter-event-interval during 5-HT application (Fig. 4c). In those neurons that responded to 5-HT application (except the one increased), bath application of 5-HT consistently reduced the frequency of mEPSCs to an average of 61.9 ± 6.0% of baseline. The inhibitory effect of 5-HT on mEPSCs frequency was reversible and showed partially recovery following washout with control ACSF (5-HT: 0.52 ± 0.05 Hz, washout 0.70 ± 0.07 Hz, *n* = 10/6, *p* < 0.05; Fig. 4a, c, e). As to the mEPSC amplitude, we did not observe evident effect of 5-HT in all recorded neurons (baseline 9.6 ± 0.6 pA; 5-HT: 8.8 ± 0.7 pA, *n* = 12/6, *p* > 0.05; Fig. 4a, b, d). These results showed that 5-HT suppressed excitatory synaptic transmission via decreasing the probability of presynaptic neurotransmitter release in most neurons.

### Postsynaptic inhibition of G proteins attenuated the reduction of EPSC caused by 5-HT

**Fig. 5:**
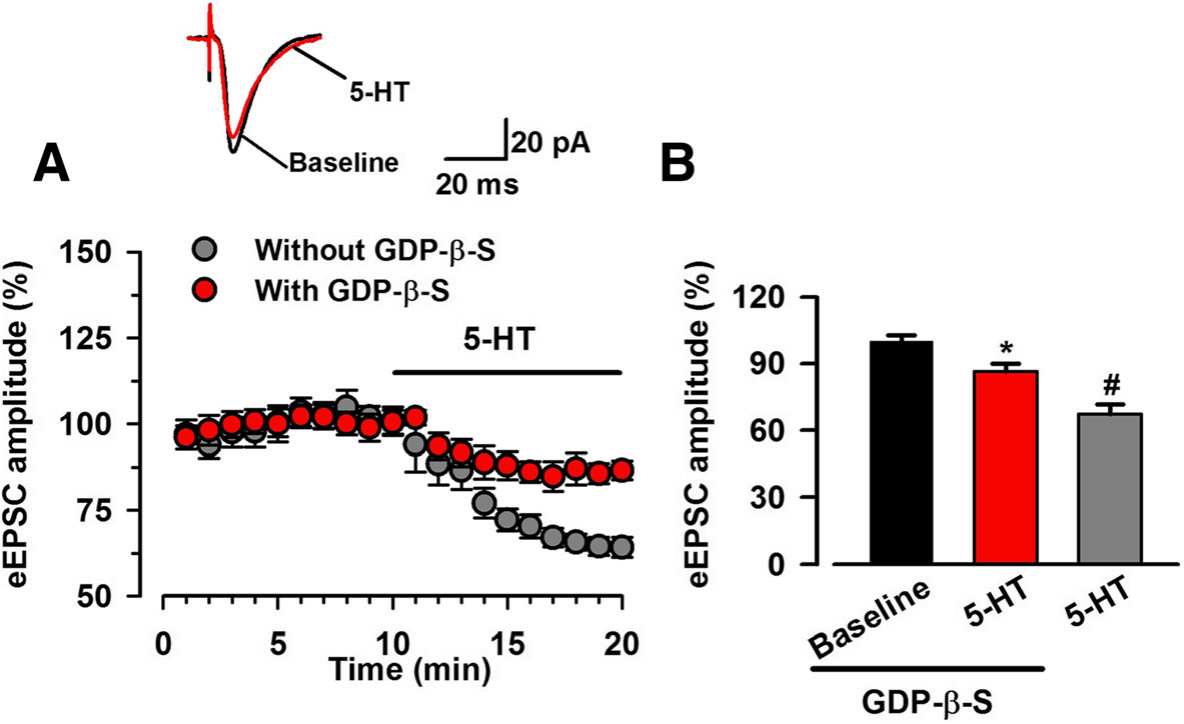
5-HT- mediated reduction of eEPSC amplitude was inhibited by GDP-β-S. **a** The averaged time course showing the change of eEPSC amplitude after bath application of 5-HT (5 μM) in the recorded ACC neurons with or without the internal solution containing GDP-β-S (1 mM). The red dots indicated the recording with GDP-β-S. The grey dots indicated the recording without GDP-β-S (data from Figure 1). **b** Summary result of effect of 5-HT on eEPSC in the presence of GDP-β-S (*n* = 9 neurons/5 mice) or absence of GDP-β-S in the recording internal solution (*n* = 9 neurons/6 mice). ^*^ *p* < 0.05 compared to baseline; ^#^ *p* < 0.05 compared to the phase of 5-HT application with GDP-β-S in the internal solution of recording electrodes

Among 5-HT receptors, only 5-HT_3_ receptor is a ligand-gated ion channel and all other subtypes are members of G-protein coupled receptors (GPCR) [25]. To investigate the role of postsynaptic 5-HT receptors in 5-HT-mediated inhibition of excitatory synaptic transmission, GDP-β-S (1 mM), a nonhydrolysable analogue of GDP that competitively inhibited G-proteins, was added to the pipette internal solution. 5-HT reduced the amplitude of evoked EPSC to a mean of 86.4 ± 3.7% of baseline in the presence of GDP-β-S (*n* = 9/5, Fig. 5), which was significantly smaller compared to the recording without GDP-β-S (Fig. 5, *p* < 0.05). This result indicated that postsynaptic 5-HT receptors were involved in the inhibitory effects of 5-HT on excitatory synaptic transmission.

### 5-HT_1A_ receptors involved in the inhibitory effect of 5-HT

**Fig. 6:**
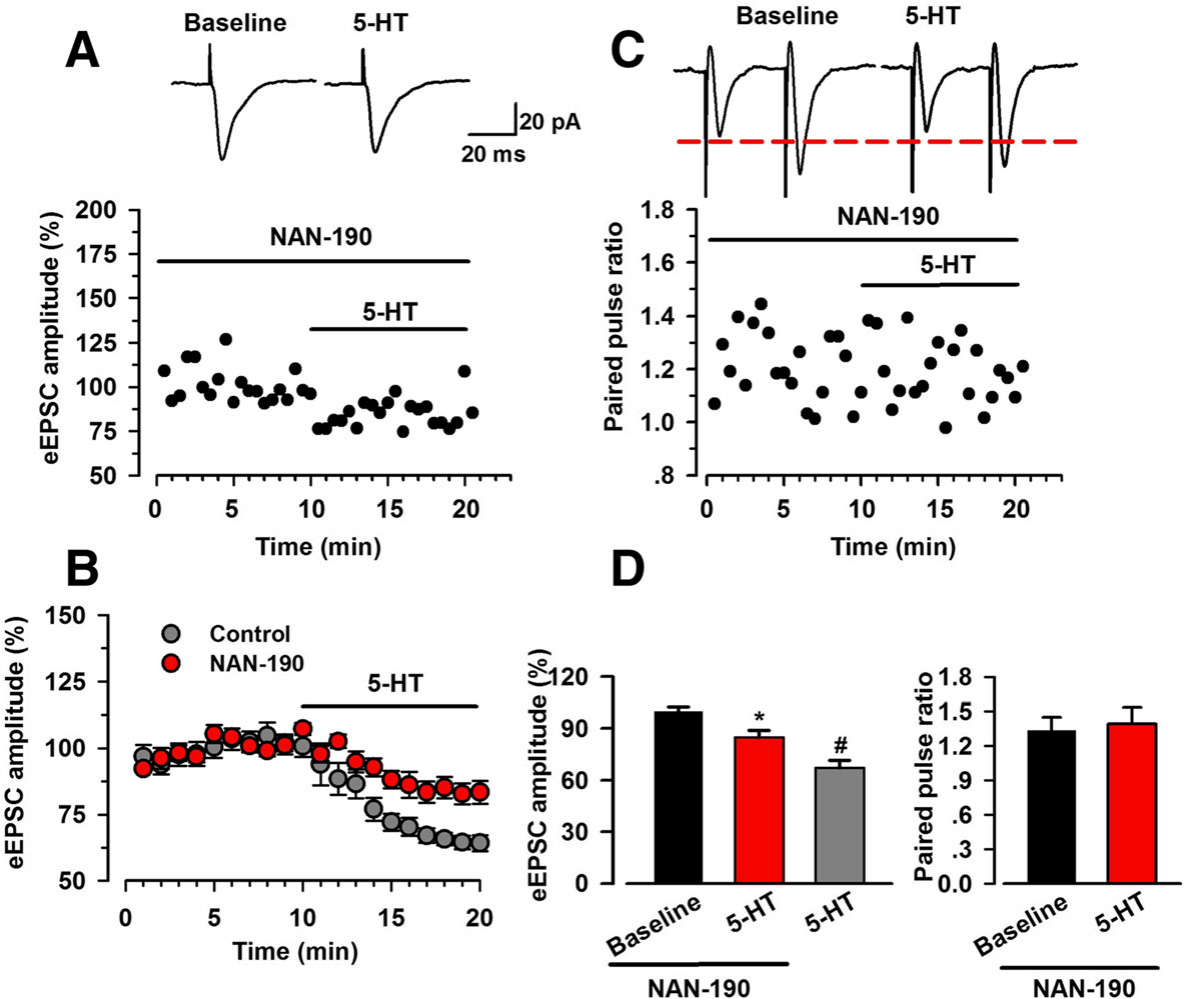
5-HT_1A_ receptor was involved in the inhibiting effect of 5-HT on synaptic transmission. **a** One sample neuron showing the time course of the change of eEPSC amplitude after 5-HT (5 μM) application in the presence of 5-HT_1A_ receptor antagonist, NAN-190 (5 μM). The insets showed averaged trace of six eEPSCs. **b** The averaged time course of recorded neurons (*n* = 9 neurons/6 mice) in ACC after application of 5-HT in the presence or absence of NAN-190. The *red* dots indicated the recording in the presence of NAN-190. The grey dots indicated the recording in the absence of NAN-190 (control, data from Figure 1). **c** One sample neuron illustrating the time course of the change of PPR after 5-HT application in the presence of NAN-190. The insets showed averaged trace of six sweeps. **d** Summary plot showing the effect of 5-HT (5 μM) on eEPSC amplitude (*left*) and PPR (*right*) when NAN-190 (5 μM) was added into the system beforehand. NAN-190 partially inhibited the reduction of eEPSC caused by 5-HT, and there was no big difference between the PPR before and after the application of 5-HT in the presence of NAN-190. *n* = 9 neurons/6 mice; ^*^ *p* < 0.05 compared to baseline. ^#^ *p* < 0.05 compared to the phase of 5-HT application in the recording with NAN-190 prior adding to the system

5-HT receptor subtypes have been identified and cloned in the CNS [26]. Previous studies report that some types of 5-HT_1_ receptors were more often associated with an inhibitory effect on excitatory synaptic transmission in certain brain regions [16, 27]. However, the role of 5-HT_1A_ receptor in the ACC has not been investigated. We bath applied NAN-190 (5 μM), one antagonist of 5-HT_1A_ receptor, to examine its effects on serotonin-mediated inhibition. We found the inhibitory effect of 5-HT on eEPSC was much smaller in the presence of NAN-190, but NAN-190 did not block the inhibitory effects of 5-HT on glutamatergic transmission (84.9 ± 4.1% of baseline, *n* = 9/6, paired *t* test, *p* < 0.05; Fig. 6a, b, d). Pretreatment with NAN-190 blocked the increase of PPR caused by 5-HT (baseline 1.33 ± 0.11, 5-HT 1.39 ± 0.15, *n* = 9/6, paired *t* test, *p* > 0.05; Fig. 6c, d).

**Fig. 7:**
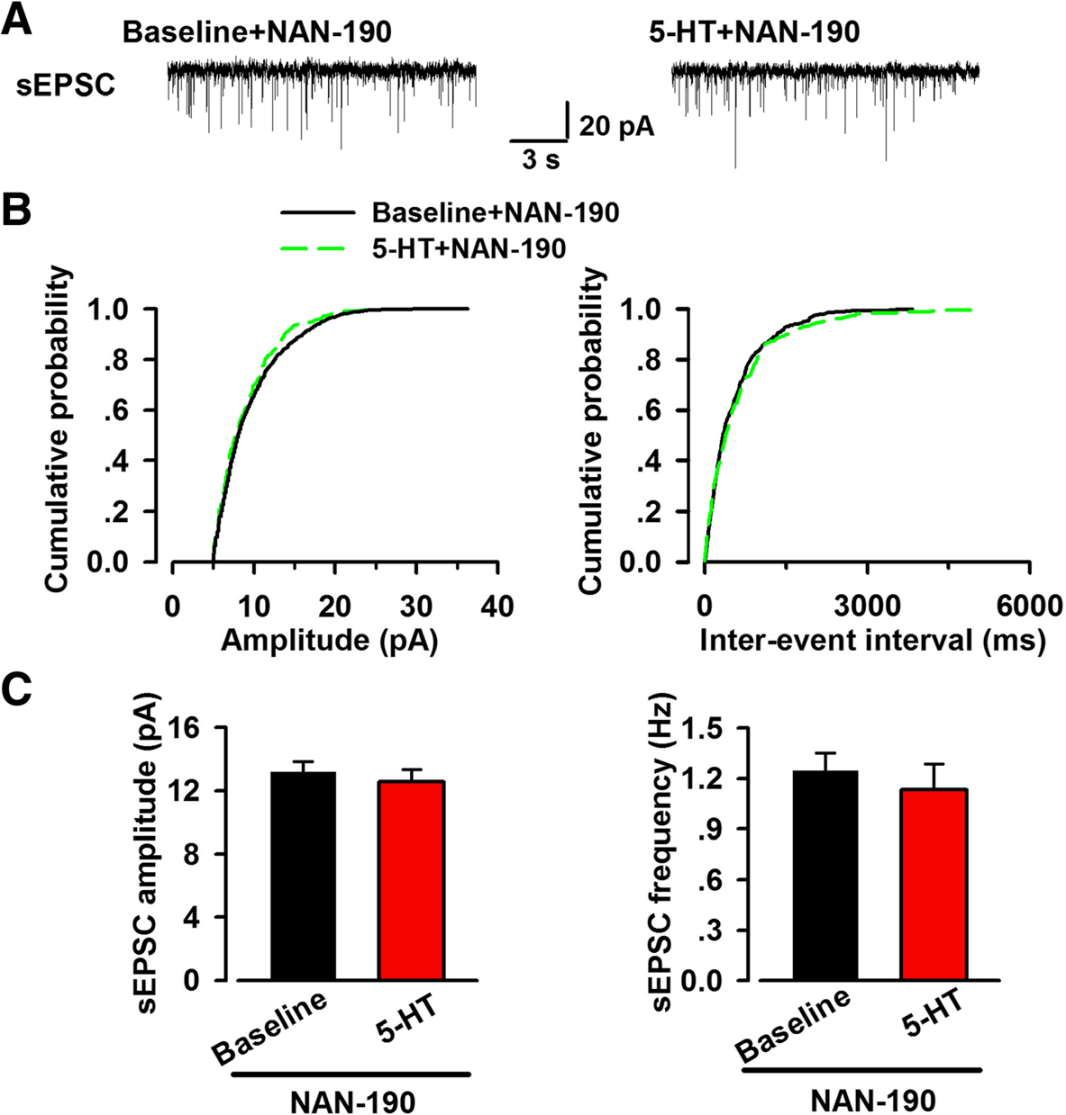
Effect of 5-HT on sEPSC in the presence of NAN-190. **a** Representative sEPSC traces recorded in the presence of NAN-190 (5 μM). **b** Cumulative probability plots showing the distribution of sEPSC amplitude (*left*) and interval-events interval (*right*) in the phase of baseline (*black line*) and 5-HT application (*dashed green line*). **c** Summary plots of sEPSC data. Averaged values of sEPSC parameters: mean amplitude (*left*) and peak frequency (*right*) (*n* = 7 neurons/5 mice). In the presence of NAN-190, 5-HT had no evident effect on the frequency and amplitude of sEPSC

**Fig. 8:**
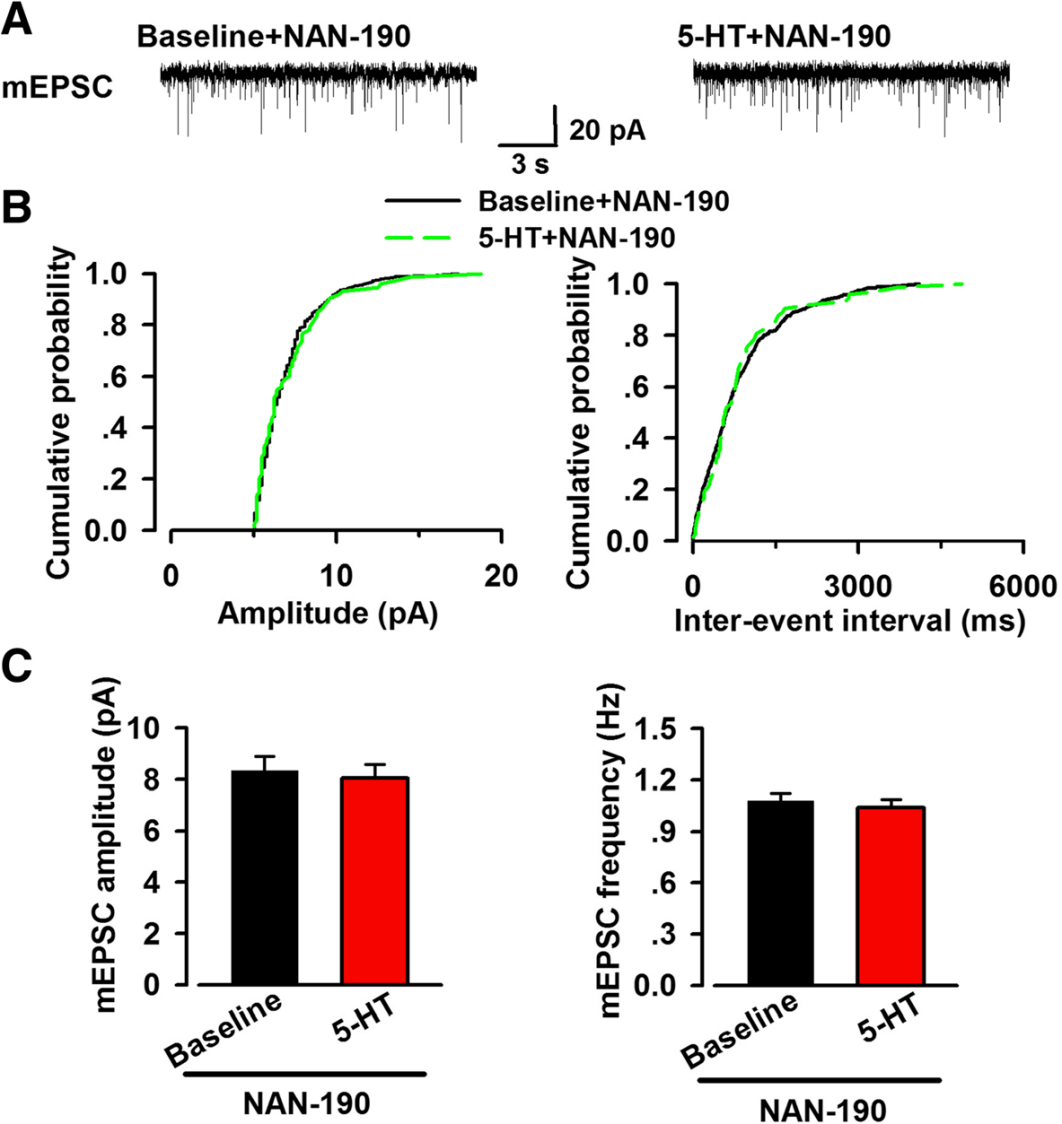
Effect of 5-HT on mEPSC in the presence of NAN-190. **a** Representative mEPSC traces recorded in the presence of NAN-190 (5 μM). **b** Cumulative probability plots showing the distribution of mEPSC amplitude (*left*) and interval-events interval (*right*). Black line represented the phase of baseline and dashed green line represented the phase of 5-HT application. **c** Summary plots of mEPSC data. Averaged values of mEPSC parameters: mean amplitude (*left*) and peak frequency (*right*) (*n* = 8 neurons/6 mice). In the presence of NAN-190, one antagonist of 5-HT_1A_ receptor, both the frequency and amplitude did not change evidently after bath application of 5-HT

Next, we examined the effects of 5-HT on sEPSC and mEPSC in the presence of NAN-190. We found 5-HT did not affect the frequency of sEPSC (baseline 1.24 ± 0.10 Hz, 5-HT 1.13 ± 0.16 Hz, *n* = 7/5, paired *t* test, *p* > 0.05; Fig. 7), as well as mEPSC (baseline 1.08 ± 0.10 Hz, 5-HT 1.04 ± 0.12 Hz, *n* = 8/6, paired *t* test, *p* > 0.05; Fig. 8) in the presence of NAN-190. These results indicated that activation of 5-HT_1A_ receptor was likely to contribute to the inhibitory effect of 5-HT on excitatory transmission in the ACC. However, the small residual effect of 5-HT in the presence of NAN-190 suggested that other 5-HT receptor subtype(s) may be involved.

## Discussion

In the present study, we tested the effects of 5-HT on synaptic transmission in the ACC. We found that 5-HT produced reversible inhibitory effects on the excitatory transmission in the ACC. 5-HT significantly decreased the frequency of sEPSC and mEPSCs and increased the PPR of eEPSCs, suggesting that 5-HT inhibited glutamate release from presynaptic terminals. Postsynaptic application of GDP-β-S reduced the inhibitory effects of 5-HT, indicating the involvement of postsynaptic 5-HT receptors in 5-HT produced inhibition. Application of 5-HT_1A_ receptors antagonist inhibited 5-HT produced inhibition of eEPSC amplitude and frequency, implying the involvement of presynaptic 5-HT_1A_ receptors in the inhibition of excitatory transmission.

### 5-HT innervation in the ACC

In the mammalian brain, 5-HT neurons can be divided into two major groups (rostral group and caudal group) based on cell body localization and their respective projections [28]. The rostral group is located in the mesencephalic and rostral pons, projecting to the forebrain, and the caudal group is located in medulla oblongata, projecting to spinal cord and brain stem [28]. The ACC receives innervation from 5-HT neurons located in the dorsal and median raphe nuclei, which belongs to rostral group [29]. There are at least fourteen different subtype receptors for 5-HT [26]. Most 5-HT receptors belong to the GPCR superfamily with the exception of 5-HT_3_ receptor, which is a ligand-gated ion channel mediating fast depolarization [25]. In the ACC, various 5-HT receptors have been detected in mouse and rat brain, such as 5-HT_1A_, 5-HT_1B_, 5-HT_2A_ and 5-HT_7_ [8, 30].

### Modulation of synaptic transmission by 5-HT

It is well known that 5-HT regulates synaptic transmission by presynaptic and/or postsynaptic mechanisms. For example, in the hippocampus, it has been reported that 5-HT inhibited glutamatergic transmission via both presynaptic and postsynaptic mechanisms [31]. In the (bed nucleus of the stria terminalis, BNST), 5-HT has been shown to suppress glutamatergic neurotransmission through activating presynaptic receptors [16]. However, there is no report of the effects of 5-HT on excitatory transmission in the ACC. In the present study, we found that 5-HT inhibited the excitatory transmission in pyramidal neurons of the ACC in a dose-dependent manner. Moreover, different experiments indicate that 5-HT may produce such inhibition through both presynaptic and postsynaptic mechanisms. In contrast to the ACC, in the spinal cord dorsal horn, a key sensory synapse for pain transmission and modulation, 5-HT produced biphasic modulation of excitatory transmission between primary afferent fibers and spinal cord dorsal horn neurons [14, 32]. These findings suggest that 5-HT modulation is different in various central synapses related to pain and/or emotion.

### Functional implications

Neurons in the ACC are involved in pain perception [1, 10, 12, 33] and emotional responses [12, 34]. Long-term plastic changes in excitatory transmission in the ACC modulate not only pain-related behaviors [35] but also the affective-emotional component of chronic pain [12, 36–38]. Especially, presynaptic potentiation of excitatory transmission in the ACC may be related pain-induced anxiety [12, 36]. Serotonergic projections are known to be important for the regulation of different brain functions, including pain and emotion [39]. Clinical drugs used for the treatment of anxiety such as selective serotonin reuptake inhibitors (SSRIs) increase the level of synaptic 5-HT. In the present study, we show that 5-HT can significantly affect excitatory transmission in the ACC. It is possible that 5-HT induced modulation in the ACC may contribute to the physiological effects of SSRIs. One of 5-HT subtype receptors is 5-HT1A. 5-HT_1A_ subtype receptors are highly expressed in the ACC [25, 40]. It has been reported that 5-HT_1A_ receptors are involved in the development of neuronal circuits regulating anxiety, and 5-HT_1A_ receptor knockout mice shows enhanced anxiety levels [41]. In our study, we found that 5-HT_1A_ mediate 5-HT induced inhibition of excitatory transmission. It is possible that changes in 5-HT_1A_ mediated modulation may contribute to chronic pain and anxiety. Future studies are clearly needed to investigate this possibility, and targeting 5-HT receptors in the ACC may provide new directions for future treatment of chronic pain and emotional disorders.
